# Supplementary material for: Ectopic Expression of PvHMA2.1 Enhances Cadmium Tolerance in Arabidopsis thaliana
Source: Int J Mol Sci. 2023 Feb 10;24(4):3544. doi: 10.3390/ijms24043544 (PMC9966247; doi:10.3390/ijms24043544)
Supplement: Supplementary file 1 [file ijms-24-03544-s001.zip › ijms-2176727-Supplementary.pdf]

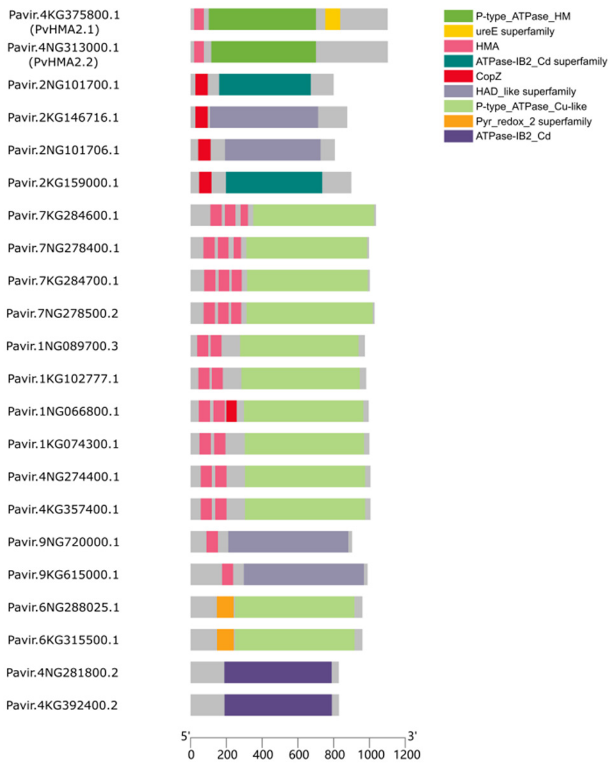

Figure S1. The conserved domains of the HMA proteins in *P. virgatum*. The conserved domains of the HMA proteins were analyzed by using Conserved Domain Search from NCBI with initial parameters. The abscissa represents the amino acid sequence length of the HMA proteins, and the boxes with different colors represent different domains in the corresponding position of each protein.

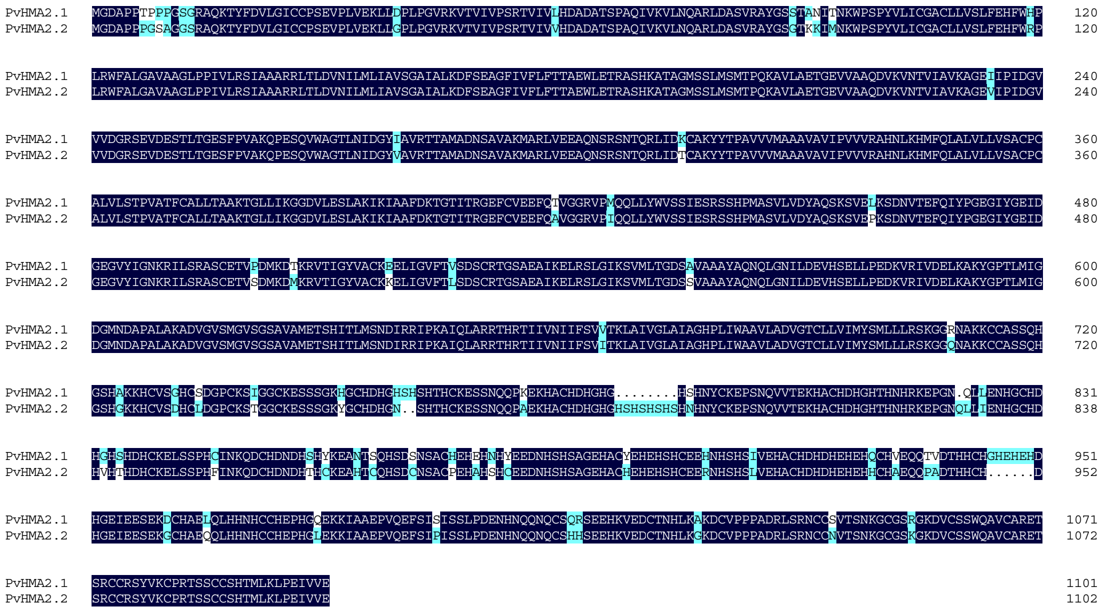

Figure S2. The amino acid sequence alignment of PvHMA2.1 and PvHMA2.2. The alignment was analyzed by using DNAMAN 6 software. The dark blue represents the identical amino acid sequence, the other colors represent different amino acid sites, and the short lines represent vacant amino acid residues.

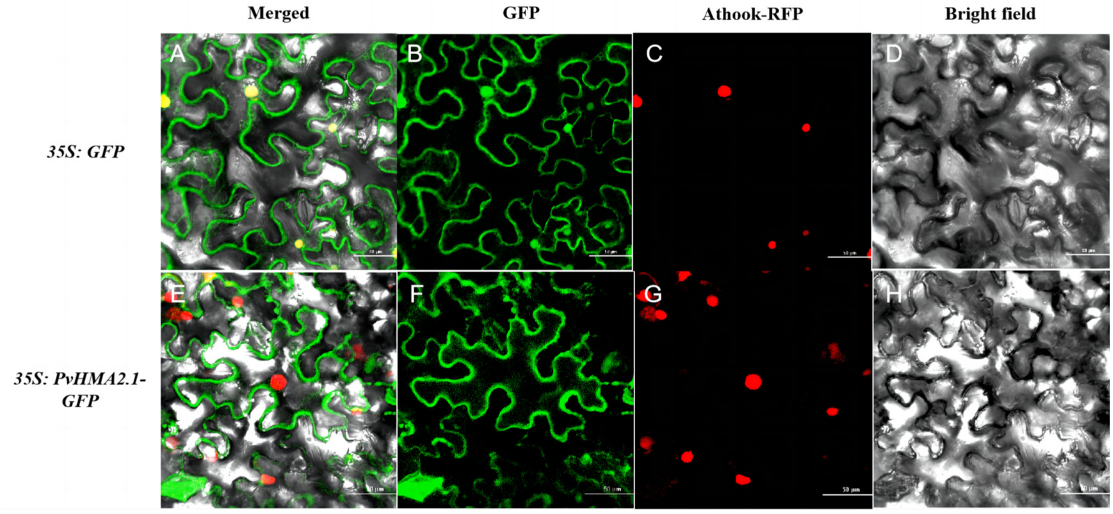

Figure S3. Subcellular localization of PvHMA2.1 in tobacco leaf cell. (**A**) The merger of GFP protein green fluorescence, the nucleus marker (Athook) protein red fluorescence, and the bright field. (**B**) The green fluorescence of GFP protein. (**E**) The merger of PvHMA2.1-GFP fusion protein green fluorescence, the nucleus marker (Athook) protein red fluorescence, and the bright field. (**F**) The green fluorescence of PvHMA2.1-GFP fusion protein. (**C**,**G**) The red fluorescence of nucleus marker (Athook) protein. (**D**,**H**) Bright field. Scale bars are 50 µm.

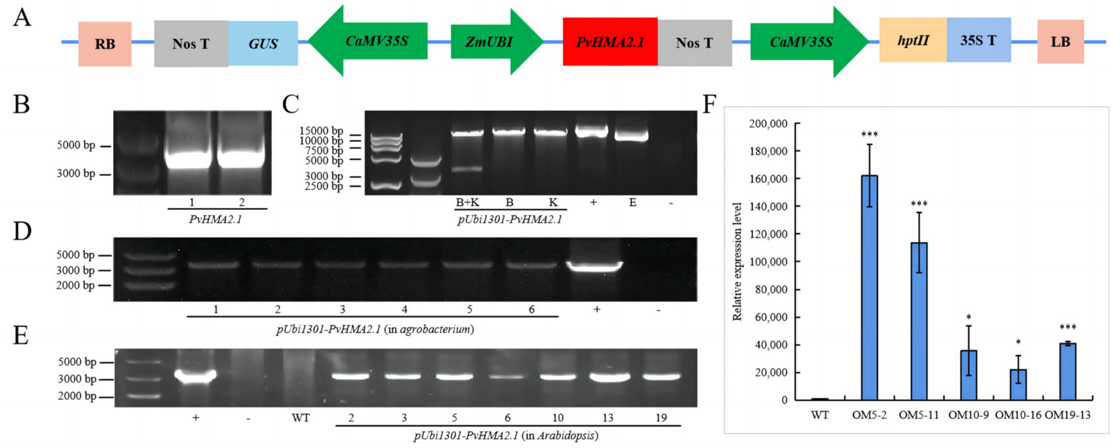

Figure S4. Ectopic expression of *PvHMA2.1* in *A. thaliana*. (**A**) The vector construction diagram of *PvHMA2.1* gene expression. Different rectangles and rectangular arrows represent different gene and promoter sequences, respectively, and the direction of the rectangular arrows represents the transcription direction. (**B**) The agarose gel electrophoretogram of *PvHMA2.1* gene in switchgrass leaves after PCR amplification. 1, 2, two biological replicates. (**C**) The agarose gel electrophoretogram of the recombinant plasmid *pUbi1301-PvHMA2.1* digestion using the restriction endonuclease. B+K, B, and K, the digestion using the restriction endonucleases of *Bam*HI and *Kpn*I, *Bam*HI only, and *Kpn*I only; E, empty plasmid *pUbi1301* without enzyme digestion; '+', the positive control of recombinant plasmid *pUbi1301-PvHMA2.1*; '-', the negative control of water. (**D**,**E**) The agarose gel electrophoretogram of *PvHMA2.1* gene after PCR amplification of recombinant plasmid *pUbi1301-PvHMA2.1* in *Agrobacterium tumefaciens* GV3101 (**D**) and wild-type *Arabidopsis* Columbia (**E**). For (**D**), 1-6, the number of monoclonal plaques in *pUbi1301-PvHMA2.1* transgenic *agrobacterium* GV3101. For (**E**), 2, 3, 5, 6, 10, 13, 19, the T_1 *PvHMA2.1* ectopically expressed *Arabidopsis* lines; WT, wild-type *Arabidopsis*. '+', the positive control of recombinant plasmid *pUbi1301-PvHMA2.1*; '-', the negative control of water. (**F**) The relative expression levels of *PvHMA2.1* in *Arabidopsis* seedlings without Cd treatment. WT, wild-type *Arabidopsis*; OM (overexpression of

PvHMA2.1)5-2, OM5-11, OM10-9, OM10-16, and OM19-13, the T3 *PvHMA2.1* ectopically expressed *Arabidopsis* lines; 7-day-old *Arabidopsis* seedlings grown on a 1/2 MS medium were harvested for RNA extraction. *Arabidopsis Actin2* expression is used as an internal reference and primers used for qPCR are listed in Supplementary Table S2. The expression levels of *PvHMA2.1* gene in WT are normalized as 1. Error bars represent the standard deviation of the three replicates. Asterisks indicate the significant differences from WT, one asterisk indicates $p < 0.05$, and three asterisks indicate $p < 0.001$.

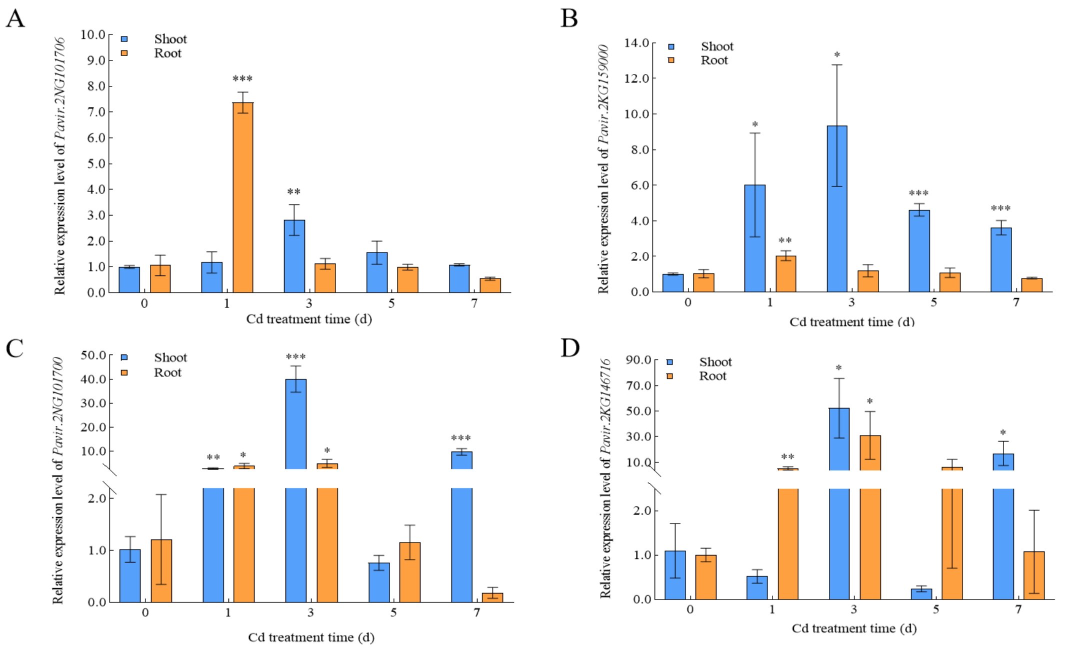

Figure S5. The relative expression levels of *Pavir.2NG101706*, *Pavir.2KG159000*, *Pavir.2NG101700*, and *Pavir.2KG146716* in response to Cd treatment. (**A-D**) The time course of *Pavir.2NG101706* (**A**), *Pavir.2KG159000* (**B**), *Pavir.2NG101700* (**C**), and *Pavir.2KG146716* (**D**) relative expression levels in switchgrass shoots and roots in response to Cd treatment. 7-day-old switchgrass seedlings were grown in a 1/4 Hoagland's nutrient solution without Cd (as the control) or with 50 μM of Cd for 7 days. The gene expression levels under the control condition are normalized as 1. Switchgrass *Ubiquitin* expression is used as an internal reference, and the primers used for the qPCR are listed in Supplementary Table S2. Error bars represent the standard deviation of the three replicates. Asterisks indicate the significant differences between the treatment group and the control group, one asterisk indicates $p < 0.05$, two asterisks indicate $p < 0.01$, and three asterisks indicate $p < 0.001$.

Table S1. Accession numbers of HMAs in *P. virgatum*, *A. thaliana*, and *O. sativa*.

Species	Accession Numbers	Sequence Names
P. virgatum	Pavir.4KG392400.2	
	Pavir.4NG281800.2	
	Pavir.4KG375800.1	PvHMA2.1
	Pavir.4NG313000.1	PvHMA2.2
	Pavir.2KG146716.1	
	Pavir.2NG101700.1	
	Pavir.2KG159000.1	
	Pavir.2NG101706.1	
	Pavir.6NG288025.1	
	Pavir.6KG315500.1	
	Pavir.9NG720000.1	

	Pavir.9KG615000.1	
	Pavir.1NG066800.1	
	Pavir.1KG074300.1	
	Pavir.1NG089700.3	
	Pavir.1KG102777.1	
	Pavir.4NG274400.1	
	Pavir.4KG357400.1	
	Pavir.7NG278500.2	
	Pavir.7KG284700.1	
	Pavir.7NG278400.1	
	Pavir.7KG284600.1	
A. thaliana	AT4G37270.1	AtHMA1
	AT4G30110.1	AtHMA2
	AT4G30120.1	AtHMA3
	AT2G19110.1	AtHMA4
	AT1G63440.1	AtHMA5
	AT4G33520.2	AtHMA6
	AT5G44790.1	AtHMA7
	AT5G21930.1	AtHMA8
O. sativa	LOC_Os06g47550.1	OsHMA1
	LOC_Os06g48720.1	OsHMA2
	LOC_Os07g12900.1	OsHMA3
	LOC_Os02g10290.1	OsHMA4
	LOC_Os04g46940.1	OsHMA5
	LOC_Os02g07630.1	OsHMA6
	LOC_Os08g37950.1	OsHMA7
	LOC_Os03g08070.1	OsHMA8
	LOC_Os06g45500.1	OsHMA9

Table S2. The list of primers used in PCR reactions.

Primer Names	Primer sequences (5'→3')	Usage
qPvHMA2.1	F: TGGACGGAGTGAGGTTGATGAGAG<br>R: GCAGAGTTGTCAGCCATAGCAGTC	qPCR
qPvHMA2.2	F: CAGTGGAACCAAAATCTGATAACG<br>R: GGCAGGAATCCGACAGAGTAAA	qPCR
qPvUbiquitin	F: CAGCGAGGGCTCAATAATTCCA<br>R: TCTGGCGGACTACAATATCCA	qPCR
PvHMA2.1-GFP	F: GGGGACAAGTTTGTACAAAAAAGCAGGCTTCATGGGGGACGCGCCGCC<br>R: GGGGACCACTTTGTACAAGAAAGCTGGGTCCTCTACCACGATCTCGGGCAG	Gene cloning
PvHMA2.1	F: ATGGGGGACGCGCCGCC<br>R: CTACTCTACCACGATCTCGGGCAG	Gene cloning
qAtActin2	F: GTCGTACAACCGGTATTGTG<br>R: GAGCTGGTCTTTGAGGTTTC	qPCR
qPavir.2NG101706	F: GAGGGGACATTCTTGAATCCTT<br>R: ATTGGGTGGCTTGATTTGCT	qPCR
qPavir.2KG159000	F: TTGAAATGAGCCAGCTTCTTTACT<br>R: GACCTTGCCATGATCCTTCTGT	qPCR
qPavir.2NG101700	F: TTCAGCATCGGTGACTTCCA<br>R: ACCTTGCCATGATCTTTCTGTTT	qPCR
qPavir.2KG146716	F: ATCAGTGCCTTCCATGTGGTT<br>R: TCCGGTCGGATAGATTTGGT	qPCR
